# Shaping Pancreatic β-Cell Differentiation and Functioning: The Influence of Mechanotransduction

**DOI:** 10.3390/cells9020413

**Published:** 2020-02-11

**Authors:** Galli Alessandra, Marku Algerta, Marciani Paola, Schulte Carsten, Lenardi Cristina, Milani Paolo, Maffioli Elisa, Tedeschi Gabriella, Perego Carla

**Affiliations:** 1 Department of Excellence of Pharmacological and Biomolecular Sciences, Università degli Studi di Milano, 20134 Milan, Italy; 2 CIMAINA, Department of Physics, Università degli Studi di Milano, 20133 Milan, Italy; 3 Department of Veterinary Medicine, Università degli Studi di Milano, 20133 Milan, Italy

**Keywords:** β-cells, mechanotransduction, diabetes, stem cells, nanotopography, islet of Langerhans, integrin, YAP/TAZ, actin

## Abstract

Embryonic and pluripotent stem cells hold great promise in generating β-cells for both replacing medicine and novel therapeutic discoveries in diabetes mellitus. However, their differentiation in vitro is still inefficient, and functional studies reveal that most of these β-like cells still fail to fully mirror the adult β-cell physiology. For their proper growth and functioning, β-cells require a very specific environment, the islet niche, which provides a myriad of chemical and physical signals. While the nature and effects of chemical stimuli have been widely characterized, less is known about the mechanical signals. We here review the current status of knowledge of biophysical cues provided by the niche where β-cells normally live and differentiate, and we underline the possible machinery designated for mechanotransduction in β-cells. Although the regulatory mechanisms remain poorly understood, the analysis reveals that β-cells are equipped with all mechanosensors and signaling proteins actively involved in mechanotransduction in other cell types, and they respond to mechanical cues by changing their behavior. By engineering microenvironments mirroring the biophysical niche properties it is possible to elucidate the β-cell mechanotransductive-regulatory mechanisms and to harness them for the promotion of β-cell differentiation capacity in vitro.

## 1. Introduction

Secreting insulin, endocrine β-cells of the pancreas are critically involved in the control of blood glucose homeostasis. Alterations of their mass or function are involved in diabetes mellitus, a pathological condition characterized by severe hyperglycemia. In type 1 diabetes mellitus, β-cell mass is lost due to an autoimmune attack, and administration of exogenous insulin is a standard therapy for these patients. In type 2 diabetes, insulin release does not compensate for the body’s needs due to β-cell dysfunction and/or insulin resistance. At late stages, decreased β-cell mass can be observed due to β-cell apoptosis or de-differentiation; at this point, only insulin administration can be effective [1,2,3]. In both cases, current therapies aim at controlling glucose levels by providing insulin, increasing insulin secretion, or improving insulin sensitivity; however, they do not regenerate β-cell mass, which is necessary to have remission. Only regenerative or replacing therapies can resolve the problem (for a review, see [4]).

Regenerating therapies such as replication from existing β-cells or trans-differentiation from other pancreatic cells can be a strategy. This feasibility has been shown in mice; however, translation of such a capacity to human cells has to be yet achieved [5,6].

Seminal works with transplanted islets provide the proof of concept that replacing strategies can work as well [7,8], and currently, 50–70% of patients who undergo islet transplantation achieve insulin independence for 5 years [9,10,11]. However, due to the paucity of human islet donors, this therapeutic option only becomes a reality for a reduced number of patients.

In vitro expansion of human β-cell lines or stem cells, once differentiated, may represent an unlimited source of β-cells for replacing strategies and pharmacological studies [12,13]. In recent years, approaches to direct the efficient differentiation of human embryonic stem cells (hESCs) and human induced pluripotent stem cells (hiPSCs) into endocrine β-cells have been developed; however, functional studies revealed that most of these β-like cells still fail to fully mirror human islet physiology, particularly in their ability to efficiently translate modifications in physiological glucose concentration into insulin release [14,15,16,17,18,19].

Teaching a cell to become a mature, efficiently secreting β-cell is not an easy task; the cell must express a variety of proteins to build up a perfect secretory apparatus able to translate alterations in blood nutrient concentrations into biochemical signals, in order to promote insulin secretion. Meanwhile, the cellular metabolic apparatus must also sustain cell activity. Currently, we are able to reproduce, in vitro, the time-dependent expression of critical transcription factors that induce β-cell differentiation, and gene profiling of ‘terminally differentiated’ stem-cell-derived β-cells provides evidence that the main proteins involved in glucose-sensing, insulin production, and secretion are expressed [20].

However, even if all the machinery is in place, the single parts must be able to crosstalk efficiently. Cells whose main function comprises secretion, like neurons, achieve high efficiency through compartmentalization of relevant molecules like receptors, channels, and downstream effectors in discrete plasma membrane domains. Although specialised membrane domains, such as dendrites and axons, are not evident in β-cells, the data on islet architecture highlight a polarized organization for these cells, with respect to their vasculature in vivo. In particular, β-cells are organized in rosette-like structures centred to a blood vessel, with three different morphological and functional domains: a small apical domain facing the central vein with the primary cilium, a lateral domain presenting the major signaling proteins involved in glucose sensing and insulin secretion, and a basal domain in contact with arterioles at the periphery [21,22,23,24,25].

From developmental studies, we know that the establishment of cellular polarity requires the presence of instructional cues delivered by the extracellular environment [26]. Traditional 2D cultures do not provide the structural conditions for the cellular organization and relationships observed in vivo. Even the perfectly functional, mature β-cell of human isolated islets de-differentiates to a non-mature phenotype soon after it is transferred into in vitro culture [27].

Conversely, the growth of stem cells in 3D structures characterized by biophysical properties and organization similar to those of the pancreas, and in vivo transplantation of stem-cell-derived immature β-cells in mice, gives rise to insulin-secreting β-cells that can restore euglycemia. This provides evidence that the in vivo environment presents cues permissive for inducing and preserving β-cell identity that until now have not yet been sufficiently reproduced in vitro [28,29,30].

Therefore, if we want to replicate in vitro what normally occurs in vivo, a full characterization and comprehension of the environmental cues of the niche, where β-cells normally develop and mature, is clearly imperative.

## 2. The Islet Niche and its Impact on β-Cell Differentiation and Function

**Figure 1:**
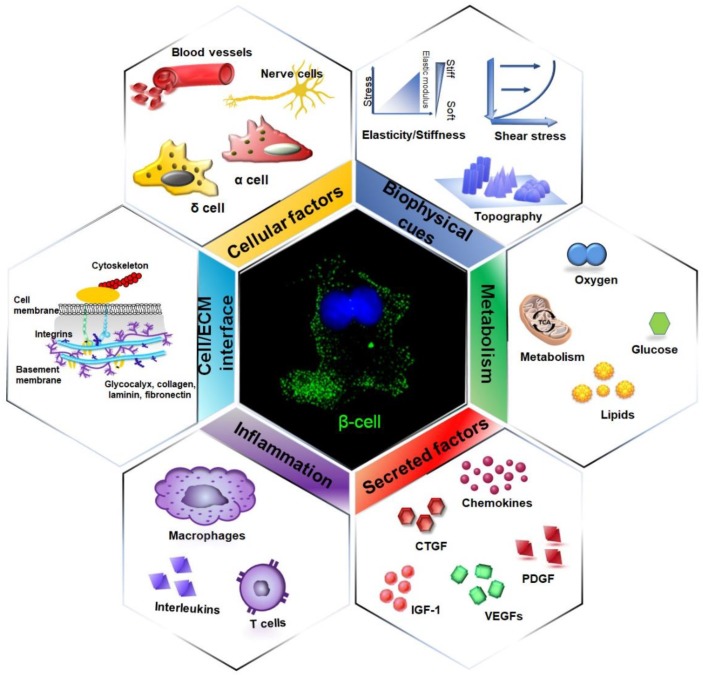
The islet niche and the extracellular factors influencing β-cell development, differentiation, and function. The islet niche is a complex and multi-factorial microenvironment that is characterized by the presence of different cells, a specific extracellular matrix, and several chemical, metabolic, and physical cues. The interactions between β-cells and their environment are extremely dynamic and bidirectional, as β-cells perceive the extracellular signals and respond to them, thus shaping the niche architecture.

Pancreatic β-cells reside in the islet niche, where they interact with other endocrine cells, vascular endothelial cells, immune and neuronal cells, and the extracellular matrix (ECM). This is a complex and dynamic microenvironment, which presents a myriad of chemical and physical stimuli that is crucial for directing β-cells differentiation (Figure 1).

β-cells communicate and integrate signals, provided by the other cells of the islets, which are essential to regulate insulin secretion and to modulate β-cell proliferation, differentiation, and mass during both development and adulthood [31,32,33,34]. The inter-endocrine cell interactions occur directly through inter-cellular junctions and indirectly through several molecules (e.g., glucagon, glutamate, and acetylcholine released by α-cells, insulin, γ-aminobutyric acid (GABA), serotonin, adenosine triphosphate (ATP), and dopamine secreted by β-cells) that exert autocrine and paracrine effects (reviewed in [35,36]).

Furthermore, β-cells are tightly interconnected with the endothelial cells of the intra-islet capillaries, which, in addition to ensuring gas exchange and nutrient supply [37], produce vascular endothelial growth factor A (VEGF-A) [38], ECM components, growth factors (e.g., insulin growth factor-IGF-1, platelet-derived growth factor-PDGF, and connective tissue growth factor-CTGF [32,39,40,41]) that are essential for β-cell development, differentiation, and function. Autonomic neurons and macrophages are also known to influence the islet niche homeostasis; neurons, indeed, fine-tune hormone secretion and regulate β-cell mass [42], while macrophages promote β-cell regeneration [43].

β-cells are also surrounded by the ECM, produced by the β-cells themselves and by the neighboring cells, which regulates multiple aspects of the islet physiology.

### 2.1. Islet ECM Composition and Structural Organization

The ECM is a three-dimensional macromolecular network that provides support for cellular attachment and stores growth factors, cytokines, and signaling molecules. It is more than just a passive support system and reservoir of macromolecules; it provides a plethora of chemical and mechanical signals that play a pivotal role in pancreas organogenesis as well as in β-cell adhesion, survival, proliferation, differentiation, and function. The ECM represents a dynamic microenvironment as its organization varies significantly between developing and mature tissues [44] and also changes in response to cellular demand. Cells perceive and respond to ECM signals shaping their activity and releasing macromolecules, which, in turn, may affect the ECM composition [45].

The islet ECM is complex and consists of a mixture of proteins, glycoproteins, proteoglycans, and glycosaminoglycans (Table 1) (reviewed in [31,45,46]).

#### 2.1.1. Laminins

A family of 15 to 20 cross-shaped trimeric glycoproteins containing α, β, and γ chains linked by disulfide bonds [47]. The ratio of laminin isoforms changes between embryonic development and adulthood; the most abundant isoforms in adult human islets are LM-511, LM-332, and LM-411, which completely replace the embryonic LM-111 [31,48]. Laminins are crucial for pancreas morphogenesis, for maintaining the integrity and the shape of the islets, as well as for the regulation of β-cell proliferation and insulin transcription [48,49].

#### 2.1.2. Collagens

Triple-helical domains proteins usually classified into fibrillar (Type I, II, III, and V) and non-fibrillar (Type IV and VI) collagens [48]. Collagens IV and VI, which form networks and beaded filaments respectively, are the most widely expressed in mature human islets of Langerhans, where they support cellular attachment and cohesiveness and regulate the ECM stiffness, which, in turn, modulates β-cell proliferation [50,51,52].

#### 2.1.3. Glycoproteins

High molecular weight macromolecules, including fibronectin and vitronectin, which are only expressed during pancreatic development [45]. These proteins contain the tripeptide recognition motif arginine–glycine–aspartic acid (RGD), which is recognized by islet cells through integrin and non-integrin receptors. This binding regulates β-cell survival and function [48].

#### 2.1.4. Glycosaminoglycans and Proteoglycans

Glycosaminoglycans are negatively charged linear sugar chains that are covalently bound to proteins (except for hyaluronic acid) to form proteoglycans. The most abundant in human islets of Langerhans is hyaluronic acid, homogenously distributed in the ECM and heparan sulphate (HS), which is preferentially concentrated in inter-cellular spaces within the islet [31,34]. HS is known to strongly affect β-cell proliferation, survival, and function, regulating the availability of signaling molecules and growth factors at the site of action [52,53].

These molecules are organized to form the complex pancreatic islet ECM macroscopic organization, characterized by several interconnected layers. An incomplete capsule, made of fibroblasts and collagen fibers, and closely associated with matrix proteins of the peri-insular basement membrane (BM), separates the islets from the exocrine tissue [45,52]. Within the islet, human endocrine cells are surrounded by two different types of BM, composed of different collagen, laminins, and fibronectin isoforms [54]: the vascular BM, which is directly associated with the endothelial cells and acts as a reservoir of growth factors [55], and the peri-islet BM, which invaginates into islets following the pervading microvasculature and can directly exchange signals with the endocrine cells [31].

### 2.2. Mechanical Cues within the Islet Niche

During development and adulthood, β-cells not only receive biochemical instructions from the extracellular environment but also experience mechanical forces, including blood shear stress, and tensile and compressing forces, which are conveyed by cell–cell and cell–matrix interactions.

The impact of mechanical cues on cell behavior has been extensively characterized during morphogenesis, when a complex interaction between intrinsic and extrinsic mechanical signals drives the formation and the growth of the developing organs [56,57]. In this section, we highlight the contribution of islet ECM stiffness, topography, and geometry, as well as the role of the fluid shear forces, to the differentiation, proliferation, and function of pancreatic β-cells.

#### 2.2.1. Stiffness

The stiffness is the ability of a ‘body’ to resist to the deformation and deflection induced by an applied force, and it is a metric of rigidity. Stiffness is measured in Pascal (Pa) [57]. In the context of mechanobiology, the stiffness is a property of the ECM and is determined by the type of collagen fibers and the level of their cross-linking. Within the body, the stiffness has been estimated to range from 0.1–10 kPa for soft tissues, such as the brain, to 1–2 GigaPascals (GPa) for hard tissues, such as bones [58]. In a seminal work of the mechanobiology field, this tissue-specific rigidity has been reported to guide mesenchymal stem cell (MSCs) lineage specification; indeed, MSCs start to express some neuronal markers when cultured on soft matrices mimicking brain stiffness, whereas osteogenic cells are obtained with stiffer matrices [59]. In addition, in soft substrates, forces can be transmitted not only between adjacent cells, but also to neighboring cells through the elastic deformation of the matrix, which strongly influences collective cell behavior [60].

Pancreatic β-cells are surrounded by the peri-islet basement membrane composed of non-fibrillar collagens with a low cross-linking rate that determines a stiffness ranging from 0.1 to 10 kPa, which classifies the pancreas as a soft tissue [61]. Since standard culture supports (plastic or glass) have a stiffness in the range of GPa [58] (much higher than that observed in the islet niche) human isolated islets of Langerhans survive poorly and lose their inter-cellular organization in in vitro cultures. Softer matrices such as hydrogels, chitosan, polylactic-coglycolid (PLGA), and poly-L-lactide (PLA) acids, which better mimic the physiological conditions, instead preserve the islet clustering organization and the β-cell function (reviewed in [54,62,63]). Indeed, when human islets are cultured on soft substrates cell–cell interactions dominate, favoring cell coalescence and preserving the cluster-like organization of the native islet; in contrast, when they are cultured on stiff supports, the extracellular-cell interactions are much stronger, causing cell scattering and the loss of islet-like structure [64].

#### 2.2.2. Topography

Topography is referred to as the distribution of surface features; in the context of the ECM we are talking about micro- and nano-environmental parameters, such as roughness, and the dimensionality and periodicity of asperities [65]. In vivo, cells are exposed to both micro- and nano-sized topographical stimuli; microscale signals (e.g., bundles of collagen fibers) can regulate collective cell behavior and migration. Beside them, the complex assembly of ECM molecules creates a nanoscale network that can influence single-cell behavior affecting cell proliferation, differentiation, and function [66,67].

To investigate the impact of such nanotopographical cues on human β-cells, our group developed nanostructured zirconia substrates with a defined ECM nanotopography-mimicking roughness by the supersonic cluster beam deposition (SCBD) technique [68,69,70], using flat featureless zirconia surfaces as a control. We demonstrated that human islets of Langerhans perceive the nanoscale properties of the substrate and activate a mechanotransductive pathway (discussed below), which promotes long-term β-cell survival and function. Interestingly, human islets grown on the nanotopographical substrates (of a certain roughness) maintain glucose-stimulated calcium currents and insulin secretion comparable to those observed in freshly isolated islet, suggesting a crucial role of nanometric topographical signals in shaping β-cell fate in in vitro cultures [64].

#### 2.2.3. Geometry

The complex organization of the cell niche, composed of several cells, vessels, nervous fibers, and ECM molecules, provides a three-dimensional environment where the cells normally reside. Each component, which has a specific nano- and microscale organization, interacts with the others, creating stiffness and topography gradients. The gradients, lacking in traditional 2D systems, can influence cell migration, cell polarity, and behavior. In addition, the 3D organization defines the type and intensity of cell–cell interactions preventing cell scattering, and thus promoting a collective response to extracellular stimuli [71]. A 3D environment is crucial for determining the spatial arrangement of pancreatic endocrine cells, which can modulate insulin secretion [46,72,73]. Indeed, in a 3D environment, β-cells are tightly interconnected among each other, promoting an extremely rapid flow of signals, which ensures efficient and coordinated insulin secretion [54,74].

#### 2.2.4. Shear Stress

Shear stress consists of the force generated by the fluid flow and exerted on the cell surface; it is mainly perceived by a primary cilium (see below), which is localised on the cell surface facing the fluid. Endothelial cells are the most obviously influenced by shear stress, reaching a magnitude of 2–4 kPa in the vessels, which induces the transcription of several genes regulating vascular homeostasis [75,76]. Even though the magnitude of this force is lower in other tissues, it plays a significant role, both during development and adulthood [58]. Human islets of Langerhans are highly vascularized, suggesting an important role of shear stress in their development and homeostasis. Indeed, blood flow, perceived by the apical cilium, regulates pancreas development and particularly β-cell polarity [77]. In addition, shear stress indirectly modulates insulin secretion by inducing the deflection of the β-cell cilium and the opening of ciliary TRPP2 (transient receptor potential nonselective cation) channels, which contribute to the maintenance of the basal levels of intracellular calcium [78].

## 3. Mechanosensing in the Islet

Although progress has been made in recent years, the molecular mechanisms by which β-cells sense and respond to mechanical cues are still largely unresolved.

The ability of the cells to perceive and discern biophysical signals provided by the extracellular environment relies on the expression of specific mechanosensors, which can be as simple as single biomolecules or as complex as multiprotein structures, adequate for the physical stimulus they receive.

### 3.1. Mechanosensors

**Figure 2:**
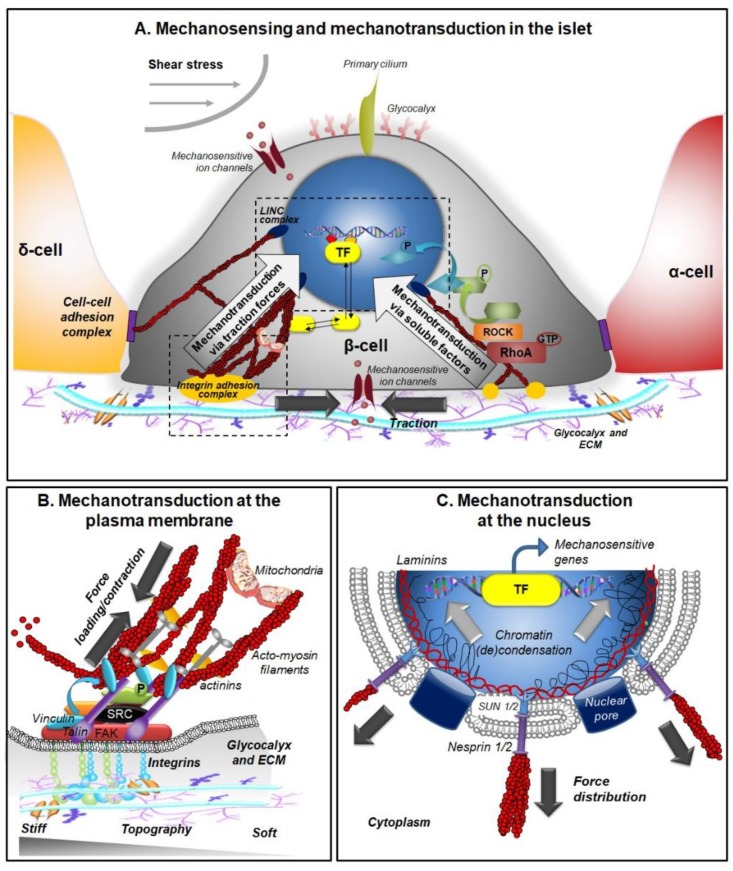
Mechanobiology in the islet (**A**) Mechanosensing and mechanotransduction in β-cells. Mechanical cues (substrate stiffness, topography and geometry, and fluid shear stress) are perceived by several mechanosensors (integrins, cadherins, mechanosensitive ion channels, primary cilia, and glycocalyx) located at the plasma membrane of β-cells. We are focusing here on integrin-based mechanotransduction. ECM physical properties are sensed by integrins and interpreted by integrin-mediated mechanotransduction, involving actin cytoskeletal actions, and the formation of integrin adhesion complexes (IAC). The latter can reach different maturation and signaling stages (from nascent adhesions, via focal complexes to focal adhesions). The dynamic composition and dimensions of IACs depend directly on the biophysical characteristics of the microenvironment that the cell encounters and can simultaneously regulate two signaling transduction pathways, differing in the timing of cellular responses. The ‘fast’ mechanoresponse (left side, time scale: milliseconds) is directly mediated by the spatial reorganization of the acto-myosin cytoskeleton, which generates modulations in tension and causes the modification of nuclear architecture and mitochondria dynamics. The slower mechanoresponse (right side, time scale: seconds to hours) is mediated by complex cascades of protein interactions and phosphorylations, which culminate with mechanosensitive transcription factors (TF, e.g., YAP/TAZ) stabilization and shuttling to the nucleus where they control gene transcription and shape the cellular program. (**B**) Mechanotransduction at the plasma membrane. The interaction of integrins with the ECM triggers the initial connection to the actin filaments (F-actin) of the cytoskeleton (via talin) and the engagement of the molecular clutch (ECM/integrin/talin/F-actin linkage) to the acto-myosin-generated forces in the nascent adhesions. The biophysical features of the ECM (in terms of the rigidity and nanometric spatial organization of the adhesion sites) determine whether these initial structures will either disintegrate, or (as depicted in the scheme) reinforce and recruit adaptor proteins (e.g., vinculin and paxillin), actin regulators (e.g., α-actinin), and signaling molecules (FAK—Focal adhesion kinase) to the nascent adhesions. This reinforcement and protein recruitment leads to a maturation of the structure (e.g., to focal adhesions) and its transformation into a signaling hub, which influences actin cytoskeletal dynamics, such as actin polymerization and the generation of acto-myosin contraction, by RhoA/ROCK pathway activation and other mechanosensitive signaling pathways (e.g., channels, YAP/TAZ). (**C**) Mechanotransduction at the nucleus. The state of cytoskeletal organization and tension, regulated by the acto-myosin fiber contraction, impacts the nuclear envelope (which is connected to the cytoskeleton via the LINC complex) and deforms its architecture and permeability to control chromatin condensation and gene activation.

Several cellular structures have been reported to act as mechanosensors, and are all expressed in β-cells: protein complexes of cell–ECM and cell–cell adhesions [60,79], primary cilia [78], stretch-activated ion channels [80], glycocalyx [81], G-protein coupled receptors [75], and growth factors receptors [82] (Figure 2A).

#### 3.1.1. Cell–Matrix and Cell–Cell Adhesion Complexes

Cell–matrix and cell–cell adhesion complexes sense the stiffness, topography, and geometry of the ECM and respond to tension and compressive forces.

A critical role in sensing these biophysical properties of the ECM is played by integrin adhesion complexes (IAC), an intricate and dynamic network of proteins made of plasma membrane adhesion receptors, adaptor proteins, actin regulators, and signaling molecules. They not only provide a physical link between the extracellular and intracellular environment but also serve as nucleation sites for signaling events that lead to changes in the cell program [83,84]. Key components of this signal transduction complex are integrins, which are heterodimeric transmembrane proteins, consisting of α and β chains, and which have regulatory and signal-transduction functions, respectively. The integrin extracellular domains bind ECM molecules, whereas the intracellular tails can be linked to the actin cytoskeleton via adaptor proteins (in particular talin), allowing force transmission. Integrins are extremely heterogeneous due to the possible combinations of 18 α- and 8 β-subunits (in mammals) and this feature ensures the binding compatibility with a large variety of ECM components, such as fibronectin, laminins, and collagens [85,86].

Human islets of Langerhans express several integrin receptors; however, the exact composition is still controversial, mainly because it is developmentally controlled. Studies report the expression of α3, α5, αv, β1, β3, and β5 integrin subunits in mature human islets, while β1 is known to play a pivotal role in regulating β-cell mass during development [45,87,88]. For comprehensive reviews of integrin composition and functions in the context of β-cell survival and differentiation see [89,90,91].

The ECM–cell interactions are also mediated by non-integrin receptors within the islets; for instance, other transmembrane receptors including the laminin receptor-1, dystroglycan complex, and Lutheran blood group glycoprotein can recognize laminins and sense extracellular mechanical stimuli [47,92]. In addition, the tyrosine kinase discoidin domain regulates ECM production and cell differentiation by binding collagen IV [93].

Cell–ECM adhesions influence cell–cell interaction complexes that regulate the force transmission between neighboring cells [94]. This inter-cellular mechanical coupling is crucial for maintaining tissue cohesiveness, ensuring a rapid flow of information among cells, which, in turn, regulates collective cell behavior [26,60,95]. Cell–cell interactions are mediated by different intercellular junctions but only adherens junctions have been recognised as mechanosensors in β-cells [61,96,97,98,99]. Adherens junctions, populated mainly by cadherins and nectins, are extremely dynamic structures that undergo conformational changes in response to mechanical stresses. Cadherins are transmembrane proteins that interact via their ectodomains and bind F-actin with the intracellular tails, creating a physical link between the actin cytoskeleton of neighboring cells. The mechanical coupling of adjacent cells is regulated by a complex interplay between cadherin ectodomains; indeed, increased tension or stress induces a cis/trans shifting of the cadherin ectodomains, and their oligomerization and clustering, which strengthens cell–cell interactions [60]. Although nectins are essential for stabilising cadherin trans interactions, their mechanosensing ability is still unknown.

#### 3.1.2. Primary Cilia

Primary cilia are short and thin structures localised at the cellular apical surface that can perceive and discern extracellular mechanical signals, especially shear stress. In 1958, Munger reported for the first time that mouse pancreatic β-cells present a primary cilium, and further studies revealed that not only human/mouse β-cells, but also α- and δ-cells possess cilia [100]. The mechanosensing ability of cilium is strictly correlated to the blood flow, which causes its deflection and the activation of intracellular signals that influence β-cell proliferation and function. The crucial role of the cilium in regulating pancreas homeostasis has been clearly demonstrated with the pancreas-specific Kinesin Family Member 3A (Kif3a) knock-out mice, which are characterized by the loss of cilia and present severe abnormalities and cyst formation within the pancreas [101]. Not only the presence but also the position of the primary cilium is important to control insulin secretion; indeed, liver kinase B1 (Lkb1) deficiency in β-cells leads to cilia relocation from the lateral membrane, closely associated with capillaries, to the opposite site, thus preventing the mechanical activation of cilia [24].

#### 3.1.3. Mechanosensitive Ion Channels

Mechanosensitive ion channels were first characterized in sensory cells where they mediate taste, vision, hearing, and also nociception [102]. In recent years, several studies have provided evidence that their mechanosensing ability is also crucial for maintaining ion homeostasis in other tissues [103,104]. The human genome codifies for 27 different transient receptor potential (TRP) channels. The mechanisms by which TRPs sense and discern mechanical stimuli are poorly understood, but several hypotheses have been raised. Tensional forces exerted by the ECM and shear stress can directly, or via the actin cytoskeleton, modulate the channel opening, thus changing the local concentration of ions. TRPs are tightly associated with other mechanosensors, such as integrins or GPCRs, which initially sense the mechanical stimuli and modify the channel activity [105].

Human pancreatic β-cells express several membrane TRPs and also the transient receptor potential polycystin 2 (TRPP2), permeable to Na^+^, K^+^, and Ca^2+^, and localized to primary cilia [106]. TRPs, by integrating a variety of extracellular stimuli, modulate the basal concentration of intracellular calcium, crucial for the regulation of glucose-stimulated insulin secretion [107]. In addition, Hayes and co-workers demonstrated that TRPs are necessary for supporting β-cell proliferation induced by PDX1 [108].

### 3.2. Mechanotransductive Processes and Signaling

Once mechanical forces and/or cues are sensed by mechanosensors, they must be translated into a biochemical signaling program that leads to alterations in gene expression and modification of cell behavior, a mechanism generally referred to as mechanotransduction (Figure 2). Cell–matrix complexes are the first to develop when the cells come into contact with a substrate and are able to sense ECM stiffness, topography, and dimensionality by generating compressive forces and force loading in the nascent adhesions [109]. Hence, we will review here mainly mechanotransduction via cell–matrix sensors. At the end of this section we will integrate, in this view, the contribution of other mechanosensors.

#### 3.2.1. Mechanotransduction at the Plasma Membrane

Integrin-based cell–matrix adhesions are prevalently involved in the response of many cells to biophysical cues [106]. Upon binding to ECM proteins, activated integrins form (via talin) an initial connection (called “molecular clutch”) to the actin cytoskeleton (F-actin) and thus to the forces generated by the so-called retrograde flow of actin (produced in combination by actin polymerization and acto-myosin contraction). This leads to a force loading within the molecular clutch, whose magnitude is determined by the biophysical properties of the microenvironment (in particular the rigidity and nanometric spatial organization of adhesion sites) that the cell interacts with [84]. In case of too (s)low or excessive force loading, the initial structure will dissociate quickly. In case of appropriate force loading, certain force thresholds in the molecular clutch will be surpassed and this causes integrin catch bond formation, talin extension, vinculin recruitment/activation, and a reinforcement of the structure. In the course of this reinforcement, integrins cluster and build up integrin adhesion complexes (IAC) by recruitment of further components such as adaptor proteins (e.g., paxillin), actin crosslinkers, and regulators (e.g., α-actinins and components of the Rho signaling machinery), signaling proteins (e.g., focal adhesion kinase (FAK) and members of Src-family of kinases) converting the IAC into a signaling platform (Figure 2B) [109,110].

Proteomic analyses indicate that integrins can interact with more than 200 known partners, with each molecule having 8–10 different potential binding partners. The resulting supramolecular complex is also called ‘integrin adhesome’ and its composition is constantly tuned by the cell biological context and the cellular microenvironment; however, a consensus adhesome has been defined [111,112]. The regulators of actin dynamics and the signaling molecules, in particular Rho GTPases, FAK, and Src, progressively change the properties of the adhesome components (e.g., through phosphorylation) and activate signal transduction pathways to control gene expression (see below). These actions lead also to a remodeling of the cytoskeleton and promotion of the generation of stress fibers. The cell contraction through these fibers is counterbalanced by the substrate stiffness at IAC and constantly tunes the cellular mechanical properties [84,113].

The formation and maturation of adhesive contacts towards focal adhesions (FA) is a complex, stepwise process driven by a continuous feedback between the progressively forming integrin adhesome and the force generated by the nascent actin fibers in response to substrate stiffness and spatial organization of integrin adhesion sites [84,113]. Generally, if the spacing of the integrin ligands is appropriate, the stiffer the substrate is, the larger and thicker the IAC and stress fibers are [84]. This holds true also for β-cells, although well-structured stress fibers are seldom observed in these cells [64].

**Figure 3:**
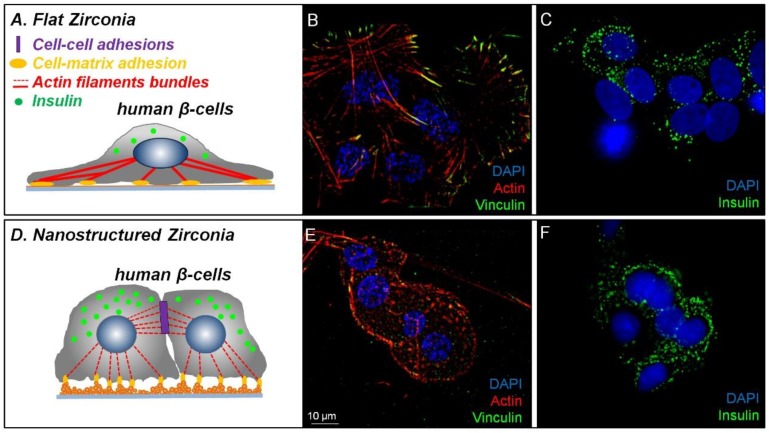
β-cells sense the ECM-mimicking nanotopography and activate a mechanotransduction-dependent program, which promotes their differentiation. Human islets of Langerhans grown on flat (**A**–**C**) or nanostructured (**D**–**F**) zirconia for 20 days were stained with DAPI (4′,6-diamidino-2-phenylindole) (blue) and for actin (red), and vinculin (green) to visualize the modulation of the mechanotransduction pathway in (**B**,**E**); or with DAPI (blue) and insulin (green) to ascertain the β-cell phenotype (**C**,**F**). (**A**–**C**) Cell–matrix interactions predominate in islet cells grown on flat substrates. β-cells present few insulin granules and are scattered on this substrate. (**D**–**F**) Cells grown on the nanotopographical substrates instead adopt a round shape, which favors the establishment of cell–cell contacts and the organization in islet-like clusters where β-cells are full of insulin granules. Note also the different nuclear shapes and sizes of the cells grown on flat or nanostructured substrates [64].

The alteration of tension generated by the cytoskeleton reorganization at cell–matrix sites also affects cell shape and the ability to form cell–cell contacts. In our experiments, when β-cells are plated on flat substrates, they develop evident FA and stress fibers and have a flat, irregular shape (Figure 3). Even if they are initially organized in clusters, over time in culture, the cells spread and tend to dissociate. β-cells on nanostructured substrates, instead develop tiny focal contacts (i.e., focal complexes) and assume a more relaxed, round shape [64]. This geometry favors the formation and maintenance of cell–cell contacts, which drives the cell organization in islet-like structures (Figure 3).

In these cells, mechanotransduction via cell–cell adhesions is prevalently mediated by proteins of the cadherin family [61]. At the cytosolic domain, their interaction triggers the recruitment of adaptor proteins to link the plasma membrane with the cytoskeleton and signaling molecules to promote the modification of the nascent adhesive junctions (AJ) and the signaling to the nucleus [95]. β-catenin is probably the most important protein of the complex, as it is involved in the formation of AJs and can also shuttle to the nucleus to control cell differentiation [114]. Cell–cell contacts also establish the lateral domain of the cells, thus defining the cell polarity. Indeed, through lateral restriction they promote the recruitment and selective retention of transporters, channels, and receptors involved in the glucose sensing and insulin secretion on this domain [22,74,115].

The generation of IAC or AJ complexes represents only the initial step of mechanotransduction, then the physical signals must be transmitted to the nucleus. Soluble regulatory factors or intracellular active tension forces modulated by the reorganization of the cytoskeleton, or a combination of both, are responsible for the final transduction of physical cues in a genetic program (Figure 2A).

#### 3.2.2. Mechanotransduction at the Nucleus via Soluble Regulatory Factors

ECM mechanosensing can be transmitted to the nucleus by regulating the shuttling of mechanotransducers and/or the activation of mechanoresponsive transcription factors (Figure 2C).

There is evidence that some FA and AJ structural proteins such as paxillin and zyxin (integrin-actin linkers) and β-catenin (cadherin-actin linker), upon mechanical force application, can shuttle to the nucleus where they can control gene activation/repression, interacting with transcription factors [116]. Little is known about paxillin and zyxin, but in β-cells β-catenin can shuttle to the nucleus and activate the T-cell factor/lymphoid enhancer-binding factor (TCF/LEF) transcription factor, which plays a key role in the control of β-cell differentiation and function [117,118].

Furthermore, signaling molecules recruited to nascent FA or AJ generate a cascade of phosphorylation events that not only control actin dynamics, but also activate transcription-relevant factors. Two transcriptional regulators clearly associated with mechanotransduction are yorkie-homologs Yes-associated protein 1 (herein YAP) and its transcriptional coactivator with PDZ-binding motif (TAZ) [119]. They bind to enhancer elements using transcriptional enhanced associate domain (TEAD) co-transcription factors and promote cell cycle progression [120]. Their activity is normally regulated by Hippo signaling through a cascade of protein phosphorylation, which causes their inactivation via cytoplasmic retention and degradation (for a review, see [121,122]).

In addition to their role as Hippo effectors, YAP/TAZ are also regulated by almost all types of mechanical stimuli [119,123]. Generally, they accumulate in the nucleus and are active in cells that experience strong mechanical forces, like cells grown on stiff substrates; conversely, they are switched-off and cytosolic in cells grown on soft substrates [119]. YAP/TAZ activation requires tension in the actomyosin cytoskeleton and FAK, Rho GTPase activity, as shown by the fact that actin depolymerizing agents, inhibitors of myosin light chain kinase, and Rho-associated protein kinase (ROCK) block mechanotransduction via YAP/TAZ [124,125]. YAP can be directly phosphorylated by Src-kinase and FAK, but whether this modification is directly involved in their activation is not yet clear [126,127].

YAP/TAZ play a key role in the mechanotransductively induced stem cell fate decision. Indeed, they are largely expressed and transcriptionally active in the nucleus of hESCs, where they foster survival and prevent differentiation, allowing for their long-term propagation. Their inhibition is instead usually required to promote stem-cell differentiation [128,129,130]. Interestingly, proteins of the Hippo signaling pathway, YAP and its downstream effectors, are present in the pancreatic lineage and YAP expression changes during pancreas development [131,132]. At early stages, YAP is highly expressed and localised mainly in the nucleus of bipotent pancreatic progenitors, where it promotes the expression of PDX1, which is crucial for sustaining their proliferation. Conversely, when differentiation of the endocrine lineages takes place, YAP loses its nuclear localization and its expression gradually decreases due to increased degradation; this favors NGN3 and NKX6.1 expression and fate commitment [131,133] (see also below).

#### 3.2.3. Mechanotransduction at the Nucleus through Cytoskeleton Tension

The anchorage of acto-myosin filaments to the plasma membrane via integrins or cadherins, and their physical link to the nuclear membrane via nesprin, a protein of the outer nuclear membrane, allow direct transmission of mechanical forces to the nucleus through the cytoskeleton [134,135,136] (Figure 2C).

The nuclear architecture is mainly controlled by the lamina, a sort of nucleoskeleton consisting of a network of intermediate-like filaments, i.e., lamin proteins, associated with the inner nuclear membrane [137]. This skeleton anchors nuclear pore complexes, which control the nuclear access to transcription–relevant factors, and participates in chromatin organization [138]. As nesprins are physically linked to lamins via proteins of the inner nuclear membrane [139,140] (LINC complex), modifications of tension at the cell periphery (due to matrix nanotopography and rigidity or cell–cell adhesion) are directly channeled to the nucleoskeleton via the cytoskeleton; this causes modifications of the nuclear architecture, possibly resulting in altered gene expression [141,142].

Two different mechanisms can therefore be envisaged to explain the effects of this long-distance force transmission on gene activation/repression. Changes in tension can modify the opening or closing of nuclear pores, thus affecting the shuttling of mechanotransducers/transcription factors between the cytoplasm and the nucleus. Alternatively, as mentioned above, the lamina also serves as the organizing centre of chromatin. Therefore, the deformation of the nucleoskeleton may alter the spatial organization of the chromatin structure and expose DNA motifs to chromatin-remodeling enzymes or transcription factors, thus regulating gene expression [136,143]. In line with the latter option, changes in histone acetylation and chromatin structure were detected in stem cells exposed to mechanical forces [144].

Interestingly, recent techniques highlight a cell-specific non-random arrangement of chromosomes within the nucleus with intermingling regions enriched in RNA polymerase II and with increased transcription activity [145,146,147]. Nuclear deformation by tension forces can modify the spatial arrangement of chromosomes and their intermingling, thus changing the cluster of neighboring genes that can be simultaneously activated [148]. This arrangement is cell–specific and can explain why similar cues produce different gene expression programs in different cell types.

We recently demonstrated that both human and clonal β-cells can activate a fast mechanotransduction pathway in response to nanotopography [64] (Figure 3). Indeed, we found reorganization of the actin cytoskeleton and modification of the nuclear shape in cells grown on nanostructured substrates with a roughness of 15 nm root-mean-square compared to flat surfaces. These data were supported by proteomic analysis, revealing the upregulation or sole expression of a huge number of nuclear structural and shuttling proteins and chromatin remodeling enzymes in the β-cells interacting with the nanotopographical surface. This indicates an important remodeling of the nuclear import/export system and chromatin condensation, promoting a gene expression program that sustains β-cell survival and differentiation [64].

Altogether, a combination of long-distance forces and soluble chemical transducers seem to drive the nuclear response of β-cells to physical cues.

### 3.3. Mechanotransduction in β-Cells: The Contribution of Metabolism

Emerging data indicate a close relationship between mechanotransduction and metabolism [149,150,151]. In response to modification of environmental cues, cells have an intrinsic ability to modulate their metabolism to better match the new energy demands. This metabolic reprogramming is quite evident during stem cell transition from self-renewal to differentiation [151] and can be the result of the specific activated gene program. However, since the shift in the metabolism often precedes cell fate establishment, it may play a permissive or instructive role on cell commitment [152,153,154].

Cell metabolism is mainly controlled by mitochondria, which provide both the energy for cell survival/proliferation and the signals for efficient glucose-dependent insulin secretion in β-cells. They are organized in dynamic networks distributed within the cells, and their shape is strictly controlled by two opposing events, fission and fusion; the first promotes the formation of small and round organelles, the latter leads to elongated, filamentous structures [155]. All the three cytoskeletal components—actin, intermediate filaments, and microtubules—interact with mitochondria; they can act as rails to support mitochondrial movement, or they can control mitochondria shape, network organization, and confinement in subdomains with high metabolic demands [156,157,158]. Therefore, it is not surprising that biophysical forces, by modifying the cytoskeletal organization, can also remodel mitochondria [153].

This has been clearly demonstrated in cardiac myocytes, where application of mechanical forces improves the maturation of myocytes and causes a modification of mitochondrial distribution and shape. Even more interesting, modification of mitochondrial organization coincides with the metabolic rewiring of myocytes [159].

Such a bi-directional relationship between mitochondrial morphology and bioenergetics has been proved in β-cells [160]. The availability of nutrients, for example, modulates mitochondrial fusion/fission and cristae architecture, which in turn controls respiratory complex assembly and function with a direct impact on the cellular metabolism [161].

We recently found that β-cell mitochondria also respond to mechanical cues. Indeed, morphological and proteomic analyses indicate modifications of the mitochondrial proteome and in their dynamics in β-cells grown on nanostructured substrates compared to β-cells on flat substrates [162]. Interestingly, the mitochondrial and nuclear modifications happen simultaneously, which suggests that they are produced by the same phenomenon (i.e., modulation of tension forces transmitted via the cytoskeleton), rather than being the result of a gene transcriptional program evoked by mechanotransduction.

In the context of β-cell differentiation, it can now be speculated that mechanical forces cause the modification of mitochondria shape and function, which leads to alterations of cellular bioenergetics and drives the final decision of cells to self-renew or differentiate. This aspect should be taken in consideration in approaches that attempt to boost β-cell differentiation with the help of biophysical cues.

## 4. Directing Pancreatic Stem Cell Differentiation by Mechanotransduction

**Figure 4:**
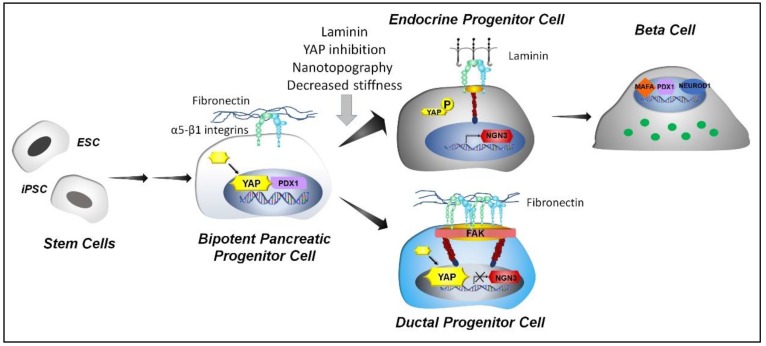
YAP signaling controls pancreatic β-cell maturation. The fate of bipotent pancreatic progenitor cells is under the control of the YAP transcription factor. Its activation promotes progenitor cell differentiation toward a ductal fate and blunt endocrinogenesis, through suppression of NGN3. Its inactivation (via modification of ECM stiffness, nanotopography, geometry, exposure to laminin, or pharmacological inhibitors) is required to allow NGN3 expression and differentiation toward an endocrine cell fate, characterized by expression of PDX1, Neurogenic differentiation 1 (NEUROD1) and MAFA transcription factors, as well as insulin.

Differentiation of stem cells toward β-cells is a long stepwise process guided by the expression of selected transcription factors. Key events are the conversion of pancreatic progenitors (expressing the PDX1 protein) to bipotent pancreatic cells that can generate both endocrine and ductal cells; their commitment toward the endocrine fate, which is marked by the expression of the transcription factor NGN3; and their final differentiation into the single-hormone-producing α, β, δ, ε, and PP cells (Figure 4). While we can efficiently induce the conversion of stem cells to endocrine precursors in vitro, the final conversion to insulin-secreting mature β-cells is still suboptimal. During development, the precise timing with which endocrine precursors located in the plexus core of the pancreatic epithelium consecutively generate α-cells, then β- and PP-cells, and finally, δ-cells [163], suggests that the endocrine fate decisions are driven by the progressive modification of their niche, in term of cell populations, chemical, and biophysical cues.

The role of mechanical cues in determining the stem cell fate toward an endocrine phenotype has only recently come into focus [164]. Although not fully explored, there is evidence that pancreatic and endocrine progenitors can sense and transduce biophysical cues into a biological program that promotes their final differentiation into mature β-cells. Very recently, different groups demonstrated that appropriate modifications of nanotopography, geometry, stiffness, and molecular composition of the extracellular environment can promote the conversion of pancreatic and endocrine progenitors toward β-cells through modulation of the YAP/TAZ signaling pathway.

Indeed, exposure of human embryonic stem cells and induced pluripotent stem cells to nanotopographical cues fosters their differentiation into pancreatic progenitors that give rise to PDX1-positive pancreatic endocrine cells. Interestingly, the increase in PDX1 expression was associated with TAZ downregulation, suggesting a potential role of TAZ in nanopatterned surface-mediated mechanotransduction [165,166].

Similarly, the epigenetic erasing and conversion of dermal fibroblasts into insulin-producing cells was promoted by growing cells on low-stiffness substrates, which prevents stress fiber formation, and modulates YAP expression and distribution within cells [167].

Recent data by Mamidi and collaborators [168] show that the physical confinement of bipotent pancreatic progenitors to a restricted area or exposure to laminin, drives their subsequent differentiation towards an endocrine lineage. Conversely, when progenitors are spread over a large area or are in contact with fibronectin, they retain a ductal fate. Mechanistic studies indicate that the process is controlled by expression of the α5 integrin and mediated by actin polymerisation and YAP-Notch mechanosignaling. When the α5 integrin is expressed (spread cells or fibronectin) the YAP-Notch axis is active; this suppresses NGN3 transcription and promotes the differentiation of bipotent pancreatic progenitors towards the duct lineage; the inhibition of the YAP-Notch mechanosignaling instead is necessary to induce endocrinogenesis.

Further evidence of the Hippo-YAP pathway involvement in the control of the endocrine cell fate comes from Rosado-Olivieri and colleagues’ work [169]. Using a combination of molecular and pharmacological approaches, they demonstrated that YAP upregulation promotes the proliferation of pancreatic precursors and blunts endocrinogenesis; YAP inhibition instead increases the conversion of progenitors to fully functional, insulin secreting β-cells. This study shows a clear involvement of YAP in defining the pancreatic progenitors’ fate, and most importantly, it provides a proof of concept that it is feasible to control the generation of mature β-cells by modifying the Hippo-YAP pathway on a pharmacological and molecular level.

While all these studies have been informative for the understanding of how pancreatic progenitors sense and respond to different biophysical inputs, the challenge now lies in systematically and quantitatively investigating the response of stem cells to diverse types, amounts, timings, frequencies, and locations of mechanotransductively relevant stimuli. Since cells are simultaneously exposed to chemical and physical cues, we should also evaluate the integrated response of stem cells to combinations of pertinent mechanical and biochemical stimuli. High-throughput, combinatorial technologies will help in screening the numerous imaginable combinations of factors that may modulate stem cell fates.

## 5. Conclusions and Perspectives

In conclusion, during development and in adult life, β-cells experience biophysical cues deriving from the ECM microenvironment (in terms of stiffness, topography, and geometry), neighboring cells, and hemodynamic shear stress. Exactly like biochemical signals, these mechanical stimuli act in a specific temporal and spatial window, and β-cells are clearly equipped with the apparatus to sense them and to respond. While many proteins or structures can function as sensors to perceive the biophysical cues at the plasma membrane level, the cytoskeleton remains the primary structure for the integration and coordination of the cellular responses.

Although we still do not know in detail the biology of their mechanotransduction, the ability of β-cells to respond to biophysical cues has already been largely exploited by tissue engineers to create in vitro cell growing supports or scaffolds for islet transplantation that better foster β-cell survival and function (excellent reviews on islets scaffolds are present in the literature, some examples are [54,62,63,170,171]).

Very recent data sustain the idea that mechanotransduction through integrins, cytoskeletal remodeling, and YAP signaling is also involved in stem-cell-derived pancreatic progenitor differentiation. Those components are probably only a part of the complex process of mechanotransduction, which we are just now starting to understand.

Although preliminary, this information supports the notion that both chemical and biophysical cues should be considered in designing the optimal niche for β-cell renewal and differentiation.

3D biomimetic scaffolds will be necessary to reproduce the complex geometry of the native niche which fosters the self-assembly of cells in clusters and the establishment of cell–cell interactions, which are essential to drive cell polarity and preserve β-cell function [172,173,174]. Nanostructured materials, possibly functionalised with relevant biomolecules, should coat the scaffold to reproduce the spatial exposition of cells to chemical and mechanical cues, which drive the decision to self-renew or differentiate [54,113,175,176]. Integration of microfluidics into the culture device will allow the reproduction of hemodynamic shear stress and the mimicking of the dynamic temporal and spatial distribution of soluble signaling factors, which is difficult to achieve with traditional cell static cultures [171,172,173,174].

Such an integrated platform has the potential to effectively boost stem cell differentiation toward cardiomyocytes [177] and could be promising also in driving the differentiation of pancreatic progenitors towards mature β-cells [178].

Even if these platforms still might not be optimal to generate huge numbers of β-cells for replacement therapy, they will certainly increase our understanding of mechanobiology in β-cells, which will be useful for designing new materials and for the discovery of novel possible targets for diabetes mellitus therapy.

## Figures and Tables

**Table 1:** ECM components involved in islet development and β-cell survival and function

ECM Component	Structure	Function	References
**Laminins**	Cross-shaped trimeric glycoproteins	Regulate pancreas morphogenesis; maintain islet shape and integrity; promote β-cell proliferation and modulate insulin transcription	[31,47,48,49]
**Collagens**	Triple-helical domain proteins, classified into fibrillar and non-fibrillar	Regulate ECM stiffness; support cell attachment and cohesiveness; promote β-cell survival	[48,50,51,52]
**Glycoproteins** *(fibronectin, vitronectin...)*	High molecular weight glycoproteins containing the tripeptide recognition motif arginine-glycine-aspartic acid (RGD)	Promote β-cell proliferation, survival and function	[45,48]
**Proteoglycans** *(e.g., heparin sulfate)*	Macromolecules composed of a core protein covalently bound to one or more glycosaminoglycans	Promote β-cell proliferation; reduce β-cell apoptosis; control postnatal islet growth and maturation	[31,34,52,53]
